# High Incidence of Metabolic Syndrome Components in Lichen Planus Patients: A Prospective Cross-Sectional Study

**DOI:** 10.1155/2022/7184678

**Published:** 2022-01-31

**Authors:** Zeinab Aryanian, Azar Shirzadian, Parvaneh Hatami, Hadiyeh Dadras

**Affiliations:** ^1^Autoimmune Bullous Diseases Research Center, Tehran University of Medical Sciences, Tehran, Iran; ^2^Department of Dermatology, Babol University of Medical Sciences, Babol, Iran

## Abstract

**Background:**

Lichen planus (LP) is a chronic inflammatory dermatosis, involving the skin, appendages, and mucous membranes. There is a growing body of evidence about higher risk of metabolic syndrome and dyslipidemia in some dermatoses including LP.

**Aim:**

To evaluate lipid profile, leptin, and CRP status among Iranian LP patients, compared to healthy controls, and peruse the relationship between abnormal values of these parameters with the disease duration and physical characteristics of patients.

**Methods:**

40 LP patients and 40 age- and sex-matched healthy controls were enrolled in the study. Data on weight, height, lipid profile, leptin, and CRP values were recorded and compared.

**Results:**

The mean values for leptin, CRP, and lipid profile parameters (except for HDL) were higher in patients, compared to controls. Total cholesterol level was negatively associated with disease duration in patients (*P* value: 0.039, r: −0.33). Serum leptin level was positively correlated with BMI both in patients and controls (*P* value: 0.037 and 0.003, respectively). In the patient group, LDL level, although insignificant, was higher in men, but HDL and leptin levels were significantly higher in women in comparison with men (*P* value: 0.018).

**Conclusion:**

Screening of LP patients in regard to their lipid profile might be more reasonable in men or those who have other cardiovascular risk factors to prevent morbidity and mortality in result of developing cardiovascular events.

## 1. Introduction

Lichen planus (LP) is a chronic inflammatory dermatosis, involving the skin, appendages, and mucous membranes. Although the exact etiopathogenesis of LP is not fully understood, current literature shows that, similar to psoriasis, a T-cell-mediated inflammatory process plays an essential role in developing the disease [1]. It is believed that the long-term release of cytokines in chronic inflammatory conditions produces metabolic derangements in terms of some disturbances in lipid/carbohydrate metabolism resulting in an increase in serum triglycerides (TG) and a decrease in high-density lipoprotein (HDL) [2, 3].

There is also a growing body of evidence about higher risk of metabolic syndrome and dyslipidemia in some skin disorders such as seborrheic dermatitis and psoriasis [4, 5].

Among the different items of metabolic syndrome, insulin resistance has been shown to be more frequently related with lichen planus [6, 7], but recently, dyslipidemia has been also reported to be associated with LP in some studies [1, 3, 8].

Leptin is a polypeptide hormone released by adipocytes and small intestine cells, regulating energy balance and body weight [9]. Previous studies have shown a relationship between leptin level and insulin resistance and obesity [10–12].

To the best of our knowledge, data on lipid profile and leptin level of patients with LP in Iran are scarce. Hence, this study was designed to evaluate patients with LP regarding their lipid profile, leptin level, and C-reactive protein (CRP) status which is one of the most important indicators of inflammatory milieu in serum.

## 2. Materials and Methods

### 2.1. Study Design

We conducted a single-center prospective cross-sectional study of adults (≥18 years of age): 40 patients diagnosed with LP clinically (with histological confirmation only in doubtful cases) who were seen at the dermatology clinic of Shahid Yahyanezhad Hospital, Babol University of Medical Sciences (BUMS), Babol, Iran, from January 2016 through March 2017 were enrolled in this study as the patient group. The control group was selected from age- and sex-matched healthy volunteers amongst companions of patients attending the out-patient dermatology clinic. The following exclusion criteria were used:

  For controls:
  Subjects with any known dermatologic or nondermatologic disorders
  Pregnant or lactating women
  Smoking habit or alcohol consumption
  For patients:
  Patients with any systemic disorder including diabetes mellitus (DM) and metabolic syndrome or any dermatologic disorder (except for LP)
  Those who received any systemic treatment for LP including corticosteroid or retinoid during last 6 months
  Pregnant or lactating women
  Smoking habit or alcohol consumption

This study was approved by the institutional ethical committee. After obtaining informed written consent from participants, all of them were subjected to a detailed review of their demographics and measurement of their weight and height to calculate the Body Mass Index (BMI) using the following formula: weight (kg)/[height (M)]^2^.

Abdominal circumference (AC) and systolic and diastolic blood pressures (SBP and DBP, respectively) were measured after a 15 min rest.

In patients, clinical type of LP was also determined and recorded.

### 2.2. Blood Sample Analysis

Blood samples were taken from participants after 12 hours of fasting. Analysis of all samples was performed at the laboratory of Shahid Yahyanezhad Hospital. Leptin level was measured using Enzyme-Linked Immunosorbent Assay (ELISA) (ME E-0300 Leptin, Germany).

We measured serum levels of CRP, total cholesterol (Chol), HDL, and Low-Density Lipoprotein (LDL) using a photometric autoanalyzer and TG with the calorimetric enzymatic method (GPO-PAP).

### 2.3. Statistical Analysis

Statistical analysis was performed using SPSS version 25 (SPSS Inc, Chicago, IL, USA). Means and standard deviations (SD) were calculated for continuous variables and frequency and percentages for categorical ones. The relationship between variables was tested using the independent *T*-test, Mann–Whitney, chi-square, and Fisher exact tests depending on the type of variables and distribution of their values. A *P* value of <0.05 was considered statistically significant, and all of the statistical analyses were performed with a 95% confidence interval (CI).

## 3. Results

### 3.1. Subject Characteristics

A total of 80 participants were enrolled in this study, 40 subjects in each group of case and control. A summary of demographics and physical characteristics of subjects is provided in Table 1.

The mean age in patient and control groups was 44.2 ± 12.4 years and 43.1 ± 9.2 years, respectively, with 50 male (24 patients and 26 controls) and 30 female (16 patients and 14 controls) subjects. There was no statistically significant difference between two groups in terms of age, sex, BMI, SBP, DBP, and AC (*P* values: 0.656, 0.500, 0.795, 0.111, 0.231, and 0.116, respectively).

Disease duration in patients was in a range of 3 months to 30 years (mean: 2.45 years)

Patients (25%) had mucosal involvement, out of which 8 had cutaneous lesions too. The remaining 30 patients (75%) had only cutaneous lesions.

### 3.2. Biochemical Assays

The leptin, CRP, Chol, TG, LDL, and HDL values for patients and controls are reported in Table 2.

As seen from the table, although mean values of all these parameters were higher in LP patients in comparison with healthy controls (except for HDL), only the CRP level intergroup difference reached to the significance level (Table 2).

Several subgroup analyses were performed to evaluate the association between participants' characteristics with parameters of this study. The results of these analyses are summarized in Table 3.

We could not find any correlation between lipid profile and age or BMI in participants. However, total cholesterol level was negatively associated with disease duration in patients (*P* value: 0.039, *r*: −0.33).

**Figure 1:**
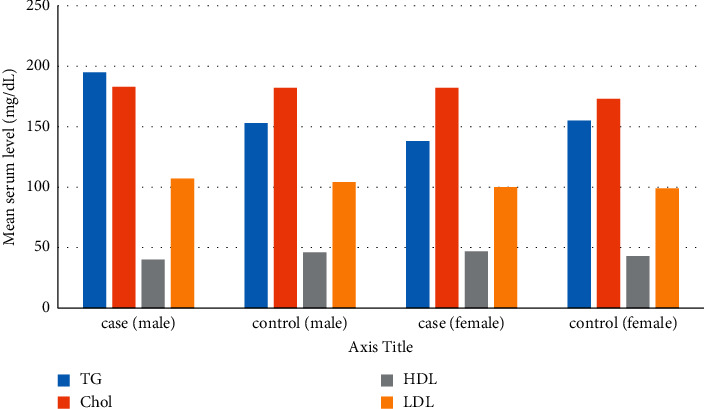
Lipid profile of participants regarding gender.

Serum leptin level was positively correlated with BMI both in patients and controls (*P* value: 0.037 and 0.003, respectively). Regarding the gender of participants, results of analyses are shown in Table 3 and Figure 1.

In the patient group, LDL level, although insignificant, was higher in men, but HDL and leptin levels were significantly higher in women in comparison with men (*P* value: 0.018).

## 4. Discussion

In the current study, we have shown that patients who have suffered from LP do not have significant dyslipidemia, compared to healthy controls. The evidence has been grown rapidly in the literature in regard to various immunologic aspects of many dermatoses and their relationship with metabolic syndrome [13–20]. In fact, higher levels of Chol, TG, and LDL have been reported in patients with LP in some studies [3, 21, 22], but the association between LP and dyslipidemia has still remained controversial [23, 24].

Our results are congruent with a meta-analysis on 4732 LP patients in which a higher level of TG, Chol, and LDL values was found in comparison with controls, but these differences did not reach the level of significance [25].

The T-cell-mediated chronic inflammatory process plays a key role in the development of LP and is thought to affect lipid/carbohydrate metabolism [2]. However, unlike other chronic dermatoses such as psoriasis or hidradenitis suppurativa, the mean duration of inflammation in LP is relatively short [2, 26]. Hence, it is more likely to find an association between dyslipidemia with long-lasting inflammatory dermatoses, compared to LP [27]. Moreover, according to Baykal et al.'s study, dyslipidemia is more frequent in LP patients with mucosal involvement [28]. However, only 22.5% of patients in our study had mucosal involvement. This may have influenced our results and biased them toward a smaller difference between controls and patients.

Hence, a relatively short duration of the inflammatory phase in LP along with less mucosal involvement in our patients, as well as possible genetic-related factors, might be considered for explaining the incongruity between our results and previous studies favoring existence of significant metabolic derangement in LP patients [3, 21, 22].

Based on the fact that prolonged dyslipidemia due to the chronic inflammatory process led to atherosclerotic plaque formation and an elevated risk of cardiovascular accidents, some studies suggested measuring of inflammatory markers such as CRP to assess cardiovascular risk [29, 30]. The inclined CRP level in patients, compared to healthy controls in our study, was noted, indicating the presence of an inflammatory process which may explain an insignificant rise in lipid profile (except for HDL) and leptin values observed in patients.

Another finding of our study was the positive association between leptin level and BMI in both patients and controls which was in agreement with other previous studies [31, 32].

It is an established fact that higher level of leptin is seen in females, compared to males [33], which was congruent with our results.

One of the interesting findings of this study was the presence of a negative correlation between total cholesterol level and disease duration. Indeed, this negative association was also noted in regards to TG and LDL values, but this correlation was significant only for Chol serum level. It can be explained by the fact that based on the established association between LP and insulin resistance and DM [6, 7], patients who have suffered from LP are advised to watch their nutrition status. This might lead to a better lipid profile over time.

Another notable finding of this study was that male patients had higher CRP level and lower HDL level, compared to female ones. Considering the protective effect of HDL against cardiovascular events [33], this may imply the greater risk for men due to lower HDL level as well as more severe inflammation. Therefore, screening of LP patients in regard to their lipid profile might be more reasonable in men or those who have other cardiovascular risk factors to prevent morbidity and mortality in result of developing cardiovascular events.

Our study had some limitations: owing to its cross-sectional design, it is not possible to detect any causal effect from its results. Small number of participants due to some financial constraints as well as rigid recruitment criteria was our another limitation.

## 5. Conclusions

In this study, we found higher mean values of lipid parameters as well as leptin level in patients with LP, compared to healthy controls, but the differences were not statistically significant. Moreover, we found lower HDL value and higher CRP level in male patients indicating probably a greater risk of cardiovascular accidents in them, compared to female patients. However, this should be further investigated in future studies.

## Figures and Tables

**Table 1:** A summary of baseline characteristics of participants in each group.

		Patient	Control	*P* value
Mean age (years)		44.2 ± 12.4	43.1 ± 9.2	0.656

Age groups, *n* (%)	≤39 years	12 (30%)	14 (35%)	0.878
	40–59 years	25 (62.5%)	24 (60%)	
	≥60 years	3 (7.5%)	2 (5%)	

Sex, *n* (%)	Male	16 (40%)	14 (35%)	0.500
	Female	24 (60%)	26 (65%)	
BMI, *n* (%)	<18.5 Kg/m^2^	1 (2.5%)	2 (5%)	0.089
	18.5–24.99 Kg/m^2^	10 (25%)	12 (30%)	
	25–29.99 Kg/m^2^	15 (37.5%)	19 (47.5%)	
	≥30 Kg/m^2^	14 (35%)	7 (17.5%)	

Mean BMI (Kg/m^2^)		27.5 ± 4.4	27.3 ± 3.2	0.795
Mean SBP (mmHg)		117 ± 12.2	112 ± 12.8	0.111
Mean DBP (mmHg)		72 ± 10.2	69 ± 9.3	0.231
Mean AC (cm)		97.5 ± 12.3	92.6 ± 15.0	0.116

Clinical type of disease, *n* (%)	Cutaneous	30 (75%)		
	Mucosal	2 (5%)		
	Mucocutaneous	8 (20%)		

BMI: body mass index, SBP: systolic blood pressure, DBP: diastolic blood pressure, AC: abdominal circumference.

**Table 2:** Biochemical parameter comparison of study participants.

	Patient	Control	*P* value
Mean CRP (mg/dl)	1.55 ± 2.17	0.61 ± 1.38	0.025
Mean TG (mg/dl)	160.4 ± 144.5	154.1 ± 150.9	0.849
Mean Chol (mg/dl)	182.6 ± 39.2	177.2 ± 36.5	0.522
Mean HDL (mg/dl)	44.2 ± 10.3	44.3 ± 6.7	0.928
Mean LDL (mg/dl)	102.7 ± 31.3	101.4 ± 27.6	0.835
Mean leptin (ng/dl)	32.6 ± 23.2	28.7 ± 20.3	0.429

CRP: c-reactive protein, TG: triglyceride, Chol: total cholesterol, HDL: high-density lipoprotein, LDL: low-density lipoprotein.

**Table 3:** *P* values and Pearson's correlations for biochemical parameters in participants regarding age, BMI, and disease duration.

		Age, *P* value (r)	BMI, *P* value (r)	Disease duration, *P* value (r)
TG (mg/dl)	Patients	0.23 (0.19)	0.44 (−0.12)	0.99 (0.11)
	Controls	0.21 (−0.2)	0.14 (0.24)	

Chol (mg/dl)	Patients	0.42 (−0.13)	0.81 (0.04)	0.39 (−0.33)
	Controls	0.33 (0.16)	0.055 (0.31)	

HDL (mg/dl)	Patients	0.27 (0.18)	0.44 (0.13)	0.98 (−0.005)
	Controls	0.07 (−0.29)	0.26 (−0.18)	

LDL (mg/dl)	Patients	0.76 (−0.05)	0.77 (−0.05)	0.054 (−0.31)
	Controls	0.45 (0.12)	0.07 (0.29)	

Leptin (ng/ml)	Patients	0.09 (−0.27)	0.037 (0.33)	0.49 (−0.11)
	Controls	0.49 (0.11)	0.003 (0.465)	

TG: triglyceride, Chol: total cholesterol, HDL: high-density lipoprotein, LDL: low-density lipoprotein.

## Data Availability

The data supporting the findings of this study are available from the corresponding author upon reasonable request.
